# Genome‐wide association for milk production and lactation curve parameters in Holstein dairy cows

**DOI:** 10.1111/jbg.12442

**Published:** 2019-10-01

**Authors:** Hadi Atashi, Mazdak Salavati, Jenne De Koster, Jim Ehrlich, Mark Crowe, Geert Opsomer, the GplusE consortium, Niamh McLoughlin, Alan Fahey, Elizabeth Matthews, Andreia Santoro, Colin Byrne, Pauline Rudd, Roisin O'Flaherty, Sinead Hallinan, Claire Wathes, Zhangrui Cheng, Ali Fouladi, Geoff Pollott, Dirk Werling, Beatriz Sanz Bernardo, Alistair Wylie, Matt Bell, Mieke Vaneetvelde, Kristof Hermans, Geert Opsomer, Sander Moerman, Jenne Koster, Hannes Bogaert, Jan Vandepitte, Leila Vandevelde, Bonny Vanranst, Johanna Hoglund, Susanne Dahl, Soren Ostergaard, Janne Rothmann, Mogens Krogh, Else Meyer, Charlotte Gaillard, Jehan Ettema, Tine Rousing, Federica Signorelli, Francesco Napolitano, Bianca Moioli, Alessandra Crisà, Luca Buttazzoni, Jennifer McClure, Daragh Matthews, Francis Kearney, Andrew Cromie, Matt McClure, Shujun Zhang, Xing Chen, Huanchun Chen, Junlong Zhao, Liguo Yang, Guohua Hua, Chen Tan, Guiqiang Wang, Michel Bonneau, Andrea Pompozzi, Armin Pearn, Arnold Evertson, Linda Kosten, Anders Fogh, Thomas Andersen, Matthew Lucey, Chris Elsik, Gavin Conant, Jerry Taylor, Nicolas Gengler, Michel Georges, Frédéric Colinet, Marilou Ramos Pamplona, Hedi Hammami, Catherine Bastin, Haruko Takeda, Aurelie Laine, Anne‐Sophie Van Laere, Martin Schulze, Sergio Palma Vera, Conrad Ferris, Cinzia Marchitelli, Miel Hostens

**Affiliations:** ^1^ Department of Reproduction, Obstetrics and Herd Health Ghent University Merelbeke Belgium; ^2^ Department of Animal Science Shiraz University Shiraz Iran; ^3^ The Roslin Institute and Royal (Dick) School of Veterinary Studies University of Edinburgh Midlothian UK; ^4^ Dairy Veterinarians Group Argyle New York; ^5^ University College Dublin Dublin Ireland

**Keywords:** genome‐wide association study, Holstein, lactation curve, milk yield

## Abstract

The aim of this study was to identify genomic regions associated with 305‐day milk yield and lactation curve parameters on primiparous (*n* = 9,910) and multiparous (*n* = 11,158) Holstein cows. The SNP solutions were estimated using a weighted single‐step genomic BLUP approach and imputed high‐density panel (777k) genotypes. The proportion of genetic variance explained by windows of 50 consecutive SNP (with an average of 165 Kb) was calculated, and regions that accounted for more than 0.50% of the variance were used to search for candidate genes. Estimated heritabilities were 0.37, 0.34, 0.17, 0.12, 0.30 and 0.19, respectively, for 305‐day milk yield, peak yield, peak time, ramp, scale and decay for primiparous cows. Genetic correlations of 305‐day milk yield with peak yield, peak time, ramp, scale and decay in primiparous cows were 0.99, 0.63, 0.20, 0.97 and −0.52, respectively. The results identified three windows on BTA14 associated with 305‐day milk yield and the parameters of lactation curve in primi‐ and multiparous cows. Previously proposed candidate genes for milk yield supported by this work include *GRINA, CYHR1, FOXH1, TONSL, PPP1R16A, ARHGAP39, MAF1, OPLAH and MROH1*, whereas newly identified candidate genes are *MIR2308, ZNF7, ZNF34, SLURP1, MAFA* and *KIFC2* (BTA14). The protein lipidation biological process term, which plays a key role in controlling protein localization and function, was identified as the most important term enriched by the identified genes.

## 1 INTRODUCTION

Milk production is mainly dependent on the shape of the lactation curve, defined as the graphical representation of milk yield over the course of the lactating period (Do et al., 2017; Ehrlich, 2011; El‐Awady, 2013). The shape of the lactation curve is characterized by the slope of the initial rise of the curve, peak yield, time to peak, the slope of the curve after peak yield (lactation persistency) and lactation length (El‐Awady, 2013; López et al., 2015; Rekik, Gara, Hamouda, & Hammami, 2003). Although all these characteristics are responsible for total milk yield per lactation, peak yield and persistency are considered as among the economically most important production traits in dairy cows (Dekkers, Ten Hag, & Weersink, 1998; Do et al., 2017; Tekerli, Akinci, Dogan, & Akcan, 2000). There is a considerable variation between animals in terms of the shape of the lactation curve, which can be attributed to factors such as genetic background, parity, diet, health status and other environmental factors (Atashi, Zamiri, & Dadpasand, 2013; Hostens, Ehrlich, Van Ranst, & Opsomer, 2012; Rekaya, Carabano, & Toro, 2000; Tekerli et al., 2000). Although several linear and non‐linear functions with different functional forms have been used to describe the relationship between daily milk yield and days in milk (DIM) in dairy cows (Sherchand, McNew, Kellogg, & Johnson, 1995; Silvestre, Petim‐Batista, & Colaco, 2006), none has achieved widespread acceptance outside a few specialized applications which are directed primarily at improving estimates of actual production from incomplete data sets (Ehrlich, 2011). The MilkBot model proposed by Ehrlich (2011) is a non‐linear lactation curve model in which parameter values can be interpreted by the effect that they have on the lactation curve. The MilkBot model is flexible enough to accommodate disease and management effect, and can provide more accurate estimates of dairy milk yield (Cole, Ehrlich, & Null, 2012).

Although genome‐wide association studies (GWAS) carried out within a variety of cattle breeds identified many genomic regions explaining variation in milk yield, they are mainly based on the polygenic estimated breeding value (EBV), daughter yield deviation (DYD) or deregressed proof for accumulated 305‐day milk yield (Cole et al., 2011; Iso‐Touru, Sahana, Guldbrandtsen, Lund, & Vilkki, 2016; Jiang et al., 2010; Meredith et al., 2012; Nayeri et al., 2016). The accumulated 305‐day milk yield is estimated by summing the test‐day milk yield (TDMY) recorded every day during the lactation period or combining the weekly or monthly TDMY by linear interpolation (Schaeffer, 2016). Since the additive genetic variance for milk yield changes during lactation (Bignardi, El Faro, Cardoso, Machado, & de Albuquerque, 2009; Singh et al., 2016), the genetic effects of QTL related to 305‐day milk yield are not constant during the lactation period; therefore, many QTL whose genetic effects change during lactation might not be detected in this approach (Lund, Sorensen, Madsen, & Jaffrézic, 2008; Ning et al., 2018). Strucken, Bortfeldt, De Koning, and Brockmann (2012) showed that lactation curve parameters provide a higher power to screen the whole genome for region whose effect change during lactation. Therefore, the objective of this study was to identify genomic region(s) associated with 305‐day milk yield and the parameters of the MilkBot lactation curve model in Holstein dairy cows.

## 2 MATERIALS AND METHODS

### 2.1 Phenotypic data

Data in this study were collected as part of Work Package 4 from the Genotype plus Environment (GplusE) FP7‐Project (http://www.gpluse.eu). The data were records of 21,068 lactations on primiparous (9,910) and multiparous (11,158) Holstein cows calving between 2010 and 2018, distributed among 118 herds in four countries (Belgium, The Netherlands, Great Britain and Denmark). To describe the lactation curve, the MilkBot model developed by Ehrlich (2011) was fitted on milk recording events from each animal. The MilkBot function is as follows:

$$y_{t}=a\left(1-\frac{\exp\left(\frac{c-t}{b}\right)}{2}\right)\exp\left(-dt\right).$$

In which, *a* is the scale parameter, representing the theoretical maximum daily yield; *b* is the ramp parameter, controlling the rate of rise in milk production in early lactation; *c* is the offset parameter, describing the offset in time between parturition and the start of lactation; and *d* is the decay parameter, representing the rate of senescence of production capacity. The time at which peak lactation occurred (*t*_peak_) was defined as: $t_{\text{peak}}=-b\ln\left(\frac{2\text{db}}{\text{db}+1}\right)+c$, and peak yield was calculated by substitution *t*_peak_ in the MilkBot equation. The 305‐day milk, the cumulative milk yield between calving and day 305 of the lactation, was calculated as:

$$M_{305}=\left(a-a\;\exp\left(-305\;d\right)\right)/d+\left(\text{ab}\;\exp\left(c/b\right)\left(-1+\exp\left(-305\left(1/b+d\right)\right)\right)\right)/\left(2+2\;\text{bd}\right).$$

### 2.2 Genotypic data

Individuals (*n* = 31,895) were genotyped using the BovineLD (*n* = 20,462), BovineSNP50K (*n* = 10,638) or BovineHD SNP panel (795 animals). Genotypes of animals were imputed to HD with a reference population of 795 (46 M and 749 F) HD individuals using FImpute V2.2 software (Sargolzaei, Chesnais, & Schenkel, 2014). In total, 12,367 out of 31,895 genotyped individuals had either phenotypic data or were in the pedigree file which was used in the association analysis (the number of animals with records was 9,910, the number of animals with records and with genotypes was 8,172, the number of animals with records and no genotypes was 1,738, and the number of animals with genotypes and no records was 4,195). Quality control (QC) was performed on the imputed data. SNP markers with minor allele frequency (MAF) less than 5% were excluded. After genomic data QC, 566,345 out of 730,539 SNP were available for the association analysis.

### 2.3 Variance components estimation

Pedigree information was collected for all phenotyped animals and contained a total of 43,181 individuals (12,367 and 9,910 out of 43,181 animals had genotype and phenotype data, respectively). The genetic analyses were carried out through the Average Information Restricted Maximum Likelihood (AIREML) method, using a linear single‐trait animal model (for measurements on the primiparous cows). The linear model included fixed effect of country and herd‐year‐season of calving, covariate effects of age at first calving in both linear and quadratic forms, and animal and residual random effects. The complete model can be represented as follows:

$$y_{\mathrm{ijk}}=\mu +{\text{HYS}}_{i}+{\text{con}}_{j}+b_{1}\left({\text{age}}_{k}\right)+b_{2}{\left({\text{age}}_{k}\right)}^{2}+a_{k}+e_{\mathrm{ijk}}$$

where *y*_ijk_ represents the response variable of animal *k*, *µ* is the overall mean, HYS_i_ is the fixed effect of *i*th herd‐year‐season of calving, con*_j_* is the fixed effect of *j*th country, *b*_1_ and *b*_2_ are the linear and quadratic regression coefficients of the dependent variable on the age at first calving, age*_k_* is the age at first calving of *k*th cows, *a_k_* is the additive genetic effect, and *e_ijk_* is the random residual error. The additive genetyic and residual variances were obtained as follows:

$$\text{var}\left[\begin{array}{c} a\\ e \end{array}\right]=\left[\begin{array}{cc} H\sigma_{a}^{2} & 0\\ 0 & I\sigma_{e}^{2} \end{array}\right]$$

where $\sigma_{a}^{2}$ and $\sigma_{e}^{2}$ are, respectively, total additive genetic and residual variances, **a** is the vector of direct additive genetic effects, **e** is a vector of residual effects, and **H** is a matrix that combines pedigree and genomic relationships, and its inverse consists on the integration of additive and genomic relationship matrices, **A** and **G**, respectively (Aguilar et al., 2010):

$$H^{-1}=A^{-1}+\left[\begin{array}{cc} 0 & 0\\ 0 & G^{-1}-A_{22}^{-1} \end{array}\right]$$

where **A** is the numerator relationship matrix based on pedigree for all animals; **A**_22_ is the numerator relationship matrix for genotyped animals; and **G** is the genomic relationship matrix which was obtained using following function described by VanRaden et al. (2009).

$$G=\frac{{\mathrm{ZDZ}}^{\prime }}{\sum_{i=1}^{M}2p_{i}\left(1-p_{i}\right)}$$

where **Z** is a matrix of gene content adjusted for allele frequencies (0, 1 or 2 for *aa*, *Aa* and *AA*, respectively); **D** is a diagonal matrix of weights for SNP variances (initially **D** = **I**); *M* is the number of SNP, and *p_i_* is the MAF of *i*th SNP. The **H** matrix was built scaling **G** based on **A_22_** considering that the average of the diagonal of **G** is equal to the average of the diagonal of **A_22_** and, the average of off‐diagonal **G** is equal to average of off‐diagonal **A_22_**. Analyses were performed using AIREMLF90 (Misztal et al., 2002). The genetic analyses for the measurements on the multiparous cows were carried out using a linear single‐trait repeatability animal model, which was the same as the model used for primiparous cows but here, the fixed effect of parity was included in the model. In addition, a third random effect representing the permanent effect associated with animals having repeated records was included in the model. This effect, assumed to be uncorrelated with additive genetic effects, allowed for the partitioning of the environmental variance into permanent and temporary components.

### 2.4 Weighted single‐step genome‐wide association study

The analyses were performed using the weighted single‐step genome‐wide association study (WssGWAS) methodology (Wang, Misztal, Aguilar, Legarra, & Muir, 2012), considering the same linear animal model used to estimate the (co) variance components mentioned before. The animal effects were decomposed into those for genotyped (**a_g_**) and ungenotyped animals (**a_n_**). The animal effects of genotyped animals are a function of the SNP effects, $a_{g}=\mathrm{Zu}$, where **Z** is a matrix relating genotypes of each locus and **u** is a vector of the SNP marker effect. The variance of animal effects was assumed as:

$$\text{Var}\;\left(a_{g}\right)=\text{Var}\;\left(\mathrm{Zu}\right)={\mathrm{ZDZ}}^{\prime }\sigma_{u}^{2}=G^{*}\sigma_{a}^{2}$$

where **D** is a diagonal matrix of weights for variances of markers (**D** = **I** for GBLUP), $\sigma_{u}^{2}$ is the genetic additive variance captured by each SNP marker when the weighted relationship matrix (G*) was built with no weight.

The SNP effects were obtained using following equation:

$$\hat{u}=\lambda {\mathrm{DZ}}^{\prime }\;G^{*-1}{\hat{a}}_{g}={\mathrm{DZ}}^{\prime }{\left[Z{\mathrm{DZ}}^{\prime }\right]}^{-1}{\hat{a}}_{g}$$

where *λ* was defined by VanRaden et al. (2009) as a normalizing constant, as described below:

$$\lambda =\frac{\sigma_{u}^{2}}{\sigma_{a}^{2}}=\frac{1}{\sum_{i=1}^{M}2p_{i}\left(1-p_{i}\right)}$$

The following iterative process described by Wang et al. (2012) was used to estimate the SNP effects. 1. **D** = **I** in the first step; 2. to calculate the **G** matrix; 3. to calculate GEBVs for the entire data set using ssGBLUP; 4. to convert GEBVs to SNP effects (*û*):$\hat{u}=\lambda {\mathrm{DZ}}^{\prime }G^{*-1}\;{\hat{a}}_{g}$; 5. to calculate the variance of each SNP:$d_{i}={\hat{u}}_{i}^{2}2p_{i}\left(1-p_{i}\right)$, where *i* is the *i*th SNP; and 6. to normalize SNP weights to keep the total genetic variance constant; exit or loop to step 2. The effects of markers were obtained by three iterations from step 2 to 6. Accuracies of GEBVs were obtained using the following formula:

$$\text{acc}=\sqrt{1-\frac{\text{PEV}}{\sigma_{g}^{2}}}$$

where PEV is the prediction error variance, and $\sigma_{g}^{2}$ is the additive genetic variance of the trait. The most accurate genomic evaluation was provided at iteration 2 which was used for estimating the percentage of genetic variance explained by *i*th genomic region as follow:

$$\frac{\text{Var(}a_{i})}{\sigma_{a}^{2}}\times 100\%=\frac{\text{Var}\left(\sum_{j=1}^{50}Z_{j}{\hat{u}}_{j}\right)}{\sigma_{a}^{2}}\times 100$$

where *a_i_* is the genetic value of the *i*th region that consists of 50 consecutive SNP, $\sigma_{a}^{2}$ is the total genetic variance, **Zj** is the vector of the SNP content of the *j*th SNP for all individuals, and ${\hat{u}}_{j}$ is the marker effect of the *j*th SNP within the *i*th region. The results were presented by the proportion of variance explained by each window of 50 consecutive SNP with an average of 165 Kb.

### 2.5 Linkage disequilibrium analysis

The square of the correlation coefficient between two loci (*r* ^2^) was used to map linkage disequilibrium (LD) using PreG SF90 (Aguilar, Misztal, Tsuruta, Legarra, & Wang, 2014) with a sliding window size of 200 Kb across the same chromosome. LD blocks were overlaid with the coordinate of the association windows as a further annotation layer.

### 2.6 Gene prospection

In a post‐GWAS study, gene ontology (GO) enrichment analysis can be performed to investigate pathways and biological processes that are shared by candidate genes related to associated regions identified in GWAS (Verardo et al., 2015). In this study, the chromosome segments that explained more than 0.50% of the additive genetic variance were selected to explore and determine potential quantitative trait loci (QTL). The Map Viewer tool of the bovine genome available at the National Center for Biotechnology Information (NCBI—http://www.ncbi.nlm.nih.gov) in the UMD3.1 bovine genome assembly and Ensembl Genome Browser (http://www.ensembl.org/index.html) was used for identification of genes. The list of genes inside the chromosome segments that explained more than 0.50% of additive genetic variance for each trait, considered as positional candidate genes, was uploaded to Enrichr for GO enrichment analysis (Chen et al., 2013; Kuleshov et al., 2016). Significantly enriched terms with at least four genes from the input gene list were identified based on the retrieved adjusted *p* value.

## 3 RESULTS

### 3.1 Variance components

Descriptive statistics for 305‐day milk yield and lactation curve parameters in both primi‐ and multiparous cows are presented in Table 1. Variance components, calculated using the AIREML method, for additive, permanent environmental (for multiparous cows) and residual variances are in Table 2. Estimated heritabilities (*SD*) were 0.37 (0.01), 0.34 (0.01), 0.17 (0.01), 0.12 (0.01), 0.30 (0.01) and 0.19 (0.01), respectively, for 305‐day milk yield, peak yield, peak time, ramp, scale and decay for primiparous cows. Genetic correlations (*SD*) of 305‐day milk yield with peak yield, peak time, ramp, scale and decay in primiparous cows were 0.99 (0.001), 0.63 (0.001), 0.20 (0.001), 0.97 (0.001) and −0.52 (0.001), respectively. Estimated heritabilities (*SD*) were 0.26 (0.02), 0.23 (0.02), 0.13 (0.02), 0.05 (0.02), 0.21 (0.02) and 0.20 (0.02), respectively, for 305‐day milk yield, peak yield, peak time, ramp, scale and decay for multiparous cows. Corresponding values for repeatability estimates were 0.42 (0.01), 0.34 (0.01), 0.15 (0.01), 0.05 (0.01), 0.29 (0.01) and 0.24 (0.01). Genetic correlations of 305‐day milk yield with peak yield, peak time, ramp, scale and decay for multiparous cows were 0.95 (0.005), 0.52 (0.005), 0.15 (0.005), 0.89 (0.005) and −0.49 (0.005), respectively.

**Table 1:** Descriptive statistics for 305‐day milk yield and lactation curve parameters^a^

Trait	Primiparous	Multiparous
	Mean (*SD*)	Mean (*SD*)
305‐day milk (kg)	8,286 (1,471)	9,966 (1990)
Ramp	30.60 (0.24)	23.27 (2.62)
Scale (kg/day)	35.57 (6.03)	49.79 (9.76)
Decay^b^	1.42 (0.40)	2.59 (0.68)
Peak time (day)	72.66 (10.48)	46.91 (8.03)
Peak yield (kg/day)	30.67 (5.26)	41.28 (7.93)

a Calculated using the following model (MilkBot model): $y_{t}=a\left(1-\frac{\exp\left(\frac{c-t}{b}\right)}{2}\right)\exp\left(-dt\right)$, in this function, *a* is the scale parameter, representing the theoretical maximum daily yield; *b* is the ramp parameter, controlling the rate of rise in milk production in early lactation; *c* is the offset parameter, describing the offset in time between parturition and the start of lactation; and *d* is the decay parameter, representing the rate of senescence of production capacity. The time at which peak lactation occurred (*t*_peak_) was defined as: $t_{\text{peak}}=-b\ln\left(\frac{2\text{db}}{\text{db}+1}\right)+c$, and peak milk production was calculated by substitution the *t*_peak_ in the MilkBot equation.

b The decay × 1,000.

**Table 2:** Variance components for 305‐ milk yield and lactation curve parameters^a^

Trait	Primiparous		Multiparous		
	$\sigma_{a}^{2}\left(\mathrm{SE}\right)$	$\sigma_{e}^{2}\left(\mathrm{SE}\right)$	$\sigma_{a}^{2}\left(\mathrm{SE}\right)$	$\sigma_{p}^{2}\left(\mathrm{SE}\right)$	$\sigma_{e}^{2}\left(\mathrm{SE}\right)$
305‐day milk (kg)	526,630 (32,046)	892,670 (21,683)	685,000 (57,726)	414,700 (49,863)	1,498,600 (34,048)
Scale (kg/day)	7.58 (0.52)	17.19 (0.39)	12.42 (1.19)	4.85 (1.11)	42.92 (0.93)
Ramp	0.0056 (0.00071)	0.0408 (0.00080)	0.3021 (0.06775)	0.0145 (0.08288)	5.6433 (0.1010)
Decay^b^	0.02228 (0.002076)	0.09256 (0.001944)	0.07120 (0.006737)	0.01143 (0.006347)	0.27119 (0.005747)
Peak time (day)	14.39 (1.43)	69.86 (1.43)	7.04 (0.86)	1.43 (0.94)	46.08 (0.94)
Peak yield (kg/day)	6.36 (0.40)	11.99 (0.28)	9.08 (0.83)	4.58 (0.75)	26.23 (0.58)

a Calculated using the following model (MilkBot model): $y_{t}=a\left(1-\frac{\exp\left(\frac{c-t}{b}\right)}{2}\right)\exp\left(-dt\right)$, in this function, *a* is the scale parameter, representing the theoretical maximum daily yield; *b* is the ramp parameter, controlling the rate of rise in milk production in early lactation; *c* is the offset parameter, describing the offset in time between parturition and the start of lactation; and *d* is the decay parameter, representing the rate of senescence of production capacity. The time at which peak lactation occurred (*t*_peak_) was defined as: $t_{\text{peak}}=-b\ln\left(\frac{2\text{db}}{\text{db}+1}\right)+c$, and peak milk production was calculated by substitution *t*_peak_ in the MilkBot equation.

b The decay × 1,000.

### 3.2 Genome‐wide association study

#### 3.2.1 Primiparous cows

**Figure 1:**
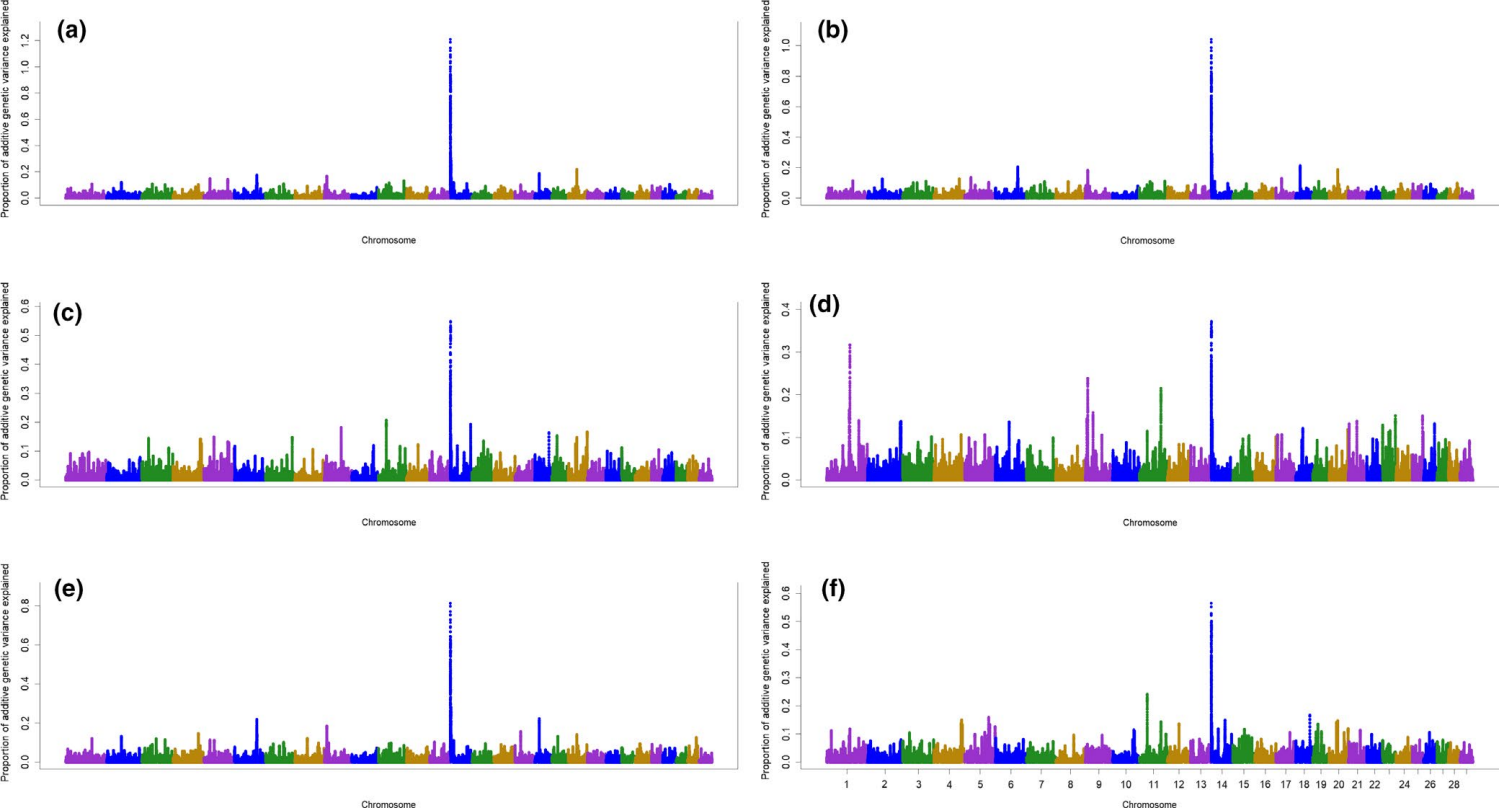
Additive genetic variance explained by windows of 50 adjacent SNP across chromosomes for 305‐day milk yield (a), peak yield (b), peak time (c), ramp (d), scale (e) and decay (f) in primiparous cows [Colour figure can be viewed at http://www.wileyonlinelibrary.com]

General information about the results of ssGWAS for primiparous cows is described in Data S1‐S6. The windows associated with 305‐day milk yield and lactation curve parameters in primiparous cows along with the genes inside them are presented in Table 3. The results identified three windows associated with the 305‐day milk yield or the parameters of the lactation curve in primiparous cows (Table 3; Figure 1). These three regions combined explained more than 3.24%, 2.80%, 1.40%, 2.18% and 1.51% of the total genetic variances of 305‐day milk yield, peak yield, peak time, scale and decay parameter of the lactation curve, respectively. However, no window was found to explain more than 0.50% of additive genetic variance of ramp. A region was found on BTA14 position 2.67–2.94 Mb which was associated with 305‐day milk yield, peak yield, peak time, scale and decay parameter of the lactation curve. A window was found on BTA14 in position 1.48–1.68 Mb which explained more than 1.21%, 1.04%, 0.81% and 0.57% of additive genetic variances of 305‐day milk yield, peak yield, scale and decay, respectively. A region on BTA14 in position 1.85–2.11 Mb was identified to be associated with 305‐day milk yield, peak yield and scale. A total of 59 genes were found to be associated with 305‐day milk yield and lactation curve parameters in primiparous cows.

**Table 3:** Identification of genes based on additive genetic variance explained by windows of 50 adjacent SNP for 305‐day milk yield and lactation curve parameters^a^ in primiparous cows

Chromosome	Position (bp)	Genes^b^	Trait (% variance explained)
BTA14	1480260–1683767	*MIR2308, LOC104973955, CYHR1, FOXH1, COMMD5, TONSL, PPP1R16A, MFSD3, LRRC24, C14H8orf33, RECQL4, ARHGAP39, RPL8, GPT, LRRC14, ZNF34, C14H8orf82, ZNF7, KIFC2*	305‐day milk (1.21), peak yield (1.04), peak time (0.47), ramp (0.37), scale (0.81), decay (0.57)
BTA14	1855090–2118405	*LOC100141215, MIR2309, MIR1839, LOC101907640, LOC101908059, LOC104968841, LOC104973958, LOC104973959, LOC104973960, LOC104973961, OPLAH, HGH1, LOC509114, GRINA, PARP10, MAF1, SHARPIN, CYC1, GPAA1, MROH1, LOC523023, EXOSC4, SPATC1, LOC786966*	305‐day milk (1.09), peak yield (0.94), peak time (0.38), ramp (0.28), scale (0.73), decay (0.44)
BTA14	2676321–2940147	*PSCA, LY6K, LOC100848939, LOC101904969, LOC101905222, LOC104973965, LOC104973966, THEM6, LYNX1, JRK, ARC, SLURP1, LY6D, GML, LOC787628, LYPD2*	305‐day milk (0.94), peak yield (0.82), peak time (0.55), ramp (0.37), scale (0.64), decay (0.50)

a Calculated using the following model (MilkBot model): $y_{t}=a\left(1-\frac{\exp\left(\frac{c-t}{b}\right)}{2}\right)\exp\left(-dt\right)$, in this function, *a* is the scale parameter, representing the theoretical maximum daily yield; *b* is the ramp parameter, controlling the rate of rise in milk production in early lactation; *c* is the offset parameter, describing the offset in time between parturition and the start of lactation; and *d* is the decay parameter, representing the rate of senescence of production capacity. The time at which peak lactation occurred (*t*_peak_) was defined as: $t_{\text{peak}}=-b\ln\left(\frac{2\text{db}}{\text{db}+1}\right)+c$, and peak milk production was calculated by substitution *t*_peak_ in the MilkBot equation.

b Official gene symbol (assembly UMD_3.1, annotation release 103).

#### 3.2.2 Multiparous cows

**Figure 2:**
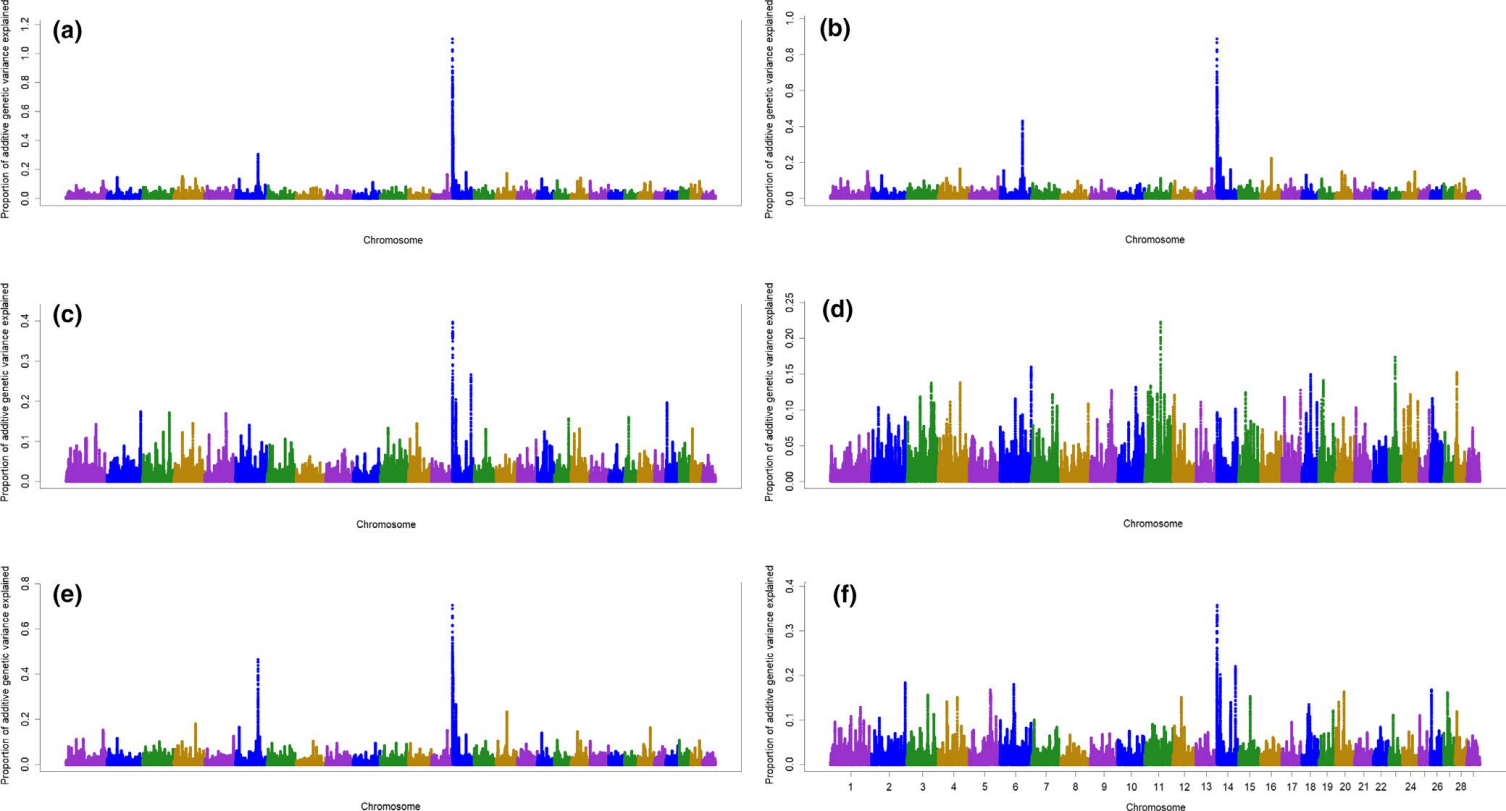
Additive genetic variance explained by windows of 50 adjacent SNP across chromosomes for 305‐day milk yield (a), peak yield (b), peak time (c), ramp (d), scale (e) and decay (f) in multiparous cows [Colour figure can be viewed at http://www.wileyonlinelibrary.com]

General information about all results of ssGWAS for multiparous cows is described in Data S7–S12. The windows associated with 305‐day milk yield and lactation curve parameters in multiparous cows along with the genes inside them are presented in Table 4. The results identified three windows associated with 305‐day milk yield and the parameters of lactation curve in multiparous cows (Table 4; Figure 2). The identified windows were quiet similar to those identified for primiparous cows. These three regions combined explained more than 2.69%, 2.18%, 0.86%, 1.75% and 0.90% of the total genetic variances of 305‐day milk yield, peak yield, peak time, scale and decay, respectively. However, no window was found to explain more than 0.50% of additive genetic variance of the peak time, decay or ramp in multiparous cows.

**Table 4:** Identification of genes based on additive genetic variance explained by windows of 50 adjacent SNP for 305‐day milk yield and lactation curve parameters^a^ in multiparous cows

Chromosome	Position (bp)	Genes^b^	Trait (% variance explained)
BTA14	1480260–1683767	*MIR2308, LOC104973955, CYHR1, FOXH1, COMMD5, TONSL, PPP1R16A, MFSD3, LRRC24, C14H8orf33, RECQL4, ARHGAP39, RPL8, GPT, LRRC14, ZNF34, C14H8orf82, ZNF7, KIFC2*	305‐day milk (1.10), peak yield (0.89), peak time (0.25), scale (0.71), decay (0.32)
BTA14	1855090–2118405	*LOC100141215, MIR2309, MIR1839, LOC101907640, LOC101908059, LOC104968841, LOC104973958, LOC104973959, LOC104973960, SPATC1, LOC786966, LOC104973961, OPLAH, HGH1, LOC509114, GRINA, PARP10, MAF1, SHARPIN, CYC1, GPAA1, MROH1, LOC523023, EXOSC4*	305‐day milk (0.88), peak yield (0.69), peak time (0.20), scale (0.56), decay (0.22)
BTA14	2676321–2940147	*PSCA, LY6K, LOC100848939, LOC101904969, LOC101905222, JRK, LYPD2, LOC104973965, LOC104973966, THEM6, LYNX1, ARC, SLURP1, LY6D, GML, LOC78762*	305‐day milk (0.79), peak yield (0.60), peak time (0.36),, scale (0.48), decay (0.36)

a Calculated using the following model (MilkBot model): $y_{t}=a\left(1-\frac{\exp\left(\frac{c-t}{b}\right)}{2}\right)\exp\left(-dt\right)$ , in this function, *a* is the scale parameter, representing the theoretical maximum daily yield; *b* is the ramp parameter, controlling the rate of rise in milk production in early lactation; *c* is the offset parameter, describing the offset in time between parturition and the start of lactation; and *d* is the decay parameter, representing the rate of senescence of production capacity. The time at which peak lactation occurred (*t*_peak_) was defined as:$t_{\text{peak}}=-b\ln\left(\frac{2\text{db}}{\text{db}+1}\right)+c$, and peak milk production was calculated by substitution *t*_peak_ in the MilkBot equation.

b Official gene symbol (assembly UMD_3.1, annotation release 103).

#### 3.2.3 Linkage disequilibrium (LD) analysis

**Figure 3:**
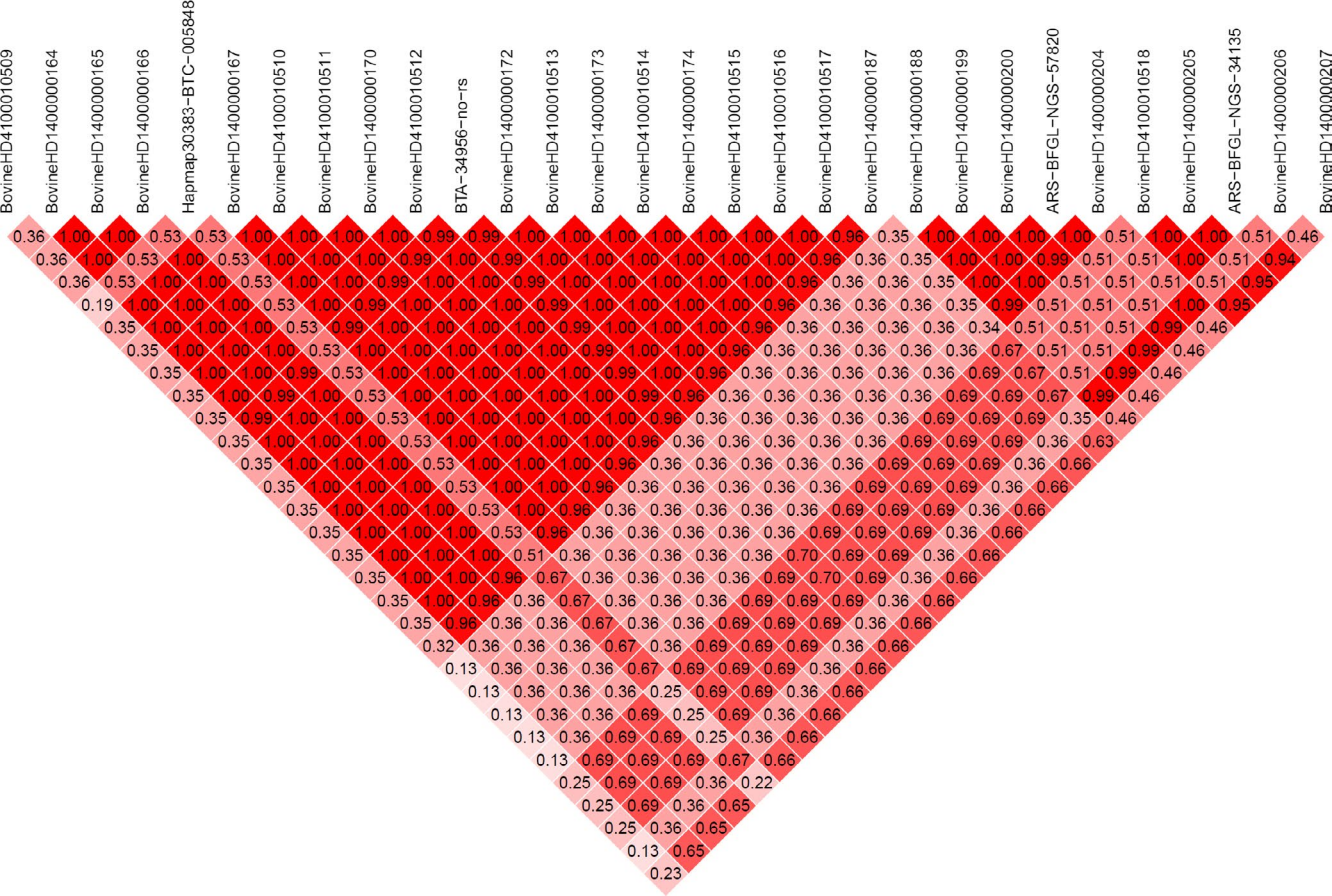
Linkage disequilibrium between 30 SNP inside the genomic region on BTA14 in position 1.48–1.68 associated with 305‐day milk and lactation curve parameters in both primiparous and multiparous cows. The colour scale ranges from red to white (colour intensity decreases with decreasing *r* ^2^ value). Strong LD was detected across a 37 kb region between SNP BovineHD1400000167 and BovineHD1400000167. This region contains genes including *ZNF7, ZNF34, RPL8, COMMD5* and *C14H8orf33* [Colour figure can be viewed at http://www.wileyonlinelibrary.com]

In total, four LD blocks consisting of 15, 5, 3 and 3 SNP were found in the region identified on BTA14 position 1.48–1.68 Mb (Figure 3), while the biggest block (across a 37 Kb region) contains genes including *ZNF7, ZNF34, RPL8, COMMD5* and *C14H8orf33*. Three LD blocks consisted of 7, 6 and 4 SNP were found in the identified region on BTA14 position 1.85–2.11 Mb, while the biggest LD block contains genes including *MIR2309* and *LOC786966*. The biggest LD block found in the window identified on BTA14 in position 2.67–2.94 Mb, consisted of nine SNP and contains genes including *LOC101905222*, *JRK* and *ARC* (results not shown).

#### 3.2.4 Gene ontology enrichment analysis

Significantly enriched biological processes with at least four genes from the input gene list are shown in Table 5. The C‐terminal protein lipidation (GO: 0006501), C‐terminal protein amino acid modification (GO: 0018410) and protein lipidation (GO: 0006497) were identified as the enriched biological processes. The protein lipidation, the covalent attachment of lipid groups to an amino acid in a protein, and C‐terminal protein amino acid modification, the alteration of the C‐terminal amino acid residue in a protein, are child terms of the C‐terminal protein lipidation, the covalent attachment of a lipid group to the carboxy‐terminus of a protein.

**Table 5:** Gene ontologies (GO) terms enriched by the genes inside the chromosomal region of associated with milk production and lactation curve parameters

Trait	GO term description	Genes
305‐day milk, Scale, peak yield, peak time, ramp	C‐terminal protein lipidation (GO:0006501)	*LY6H, LY6K, LY6D, LYPD2, PSCA, GPIHBP1*
305‐day milk, scale, peak yield, peak time, ramp	C‐terminal protein amino acid modification (GO:0018410)	*LY6H, LY6K, LY6D, LYPD2, PSCA, GPIHBP1*
305‐day milk, scale, peak yield, peak time, ramp	protein lipidation (GO:0006497)	*LY6H, LY6K, LY6D, LYPD2, PSCA, GPIHBP1*

## 4 DISCUSSION

The heritability of 305‐day milk yield was 0.37 and 0.26, respectively, in primiparous and multiparous cows. The heritability for lactation curve parameters in primiparous cows ranged from 0.12 (ramp) to 0.34 (peak yield). The corresponding values in multiparous cows were 0.05–0.23, respectively, for ramp and peak yield. Previous researchers have reported that peak yield is more heritable compared with other parameters of the lactation curve (Gebreyohannes, Koonawootrittriron, Elzo, & Suwanasopee, 2013; Saghanezhad, Atashi, Dadpasand, Zamiri, & Shokri‐Sangari, 2017; Shanks, Berger, Freeman, & Dickinson, 1981). Genetic correlations of 305‐day milk yield with lactation curve parameters ranged from −0.52 (decay) to 0.99 (peak yield). The corresponding values in multiparous cows were −0.49 to 0.95, respectively, for decay and peak yield. The higher correlation between 305‐day milk yield and peak yield compared to decay, as an indicator for lactation persistency, indicates that peak yield is more important in determining the lactation yield than persistency and can be used as a management tool to monitor milk production performance of the herd (Ali & Schaeffer, 1987; Atashi, Zamiri, & Sayyadnejad, 2012; Gebreyohannes et al., 2013; Tekerli et al., 2000).

In this study, the weighted single‐step genomic BLUP (WssGWAS) approach described by Wang et al. (2012) was used to identify genomic region(s) associated with 305‐day milk yield and lactation curve parameters in Holstein dairy cows. The WssGWAS approach integrates all phenotypic, genotypic and pedigree data simultaneously; therefore, there is no need to calculate pseudo‐phenotypes. In addition, this approach allows the use of different weights for SNP according to their importance, which is a deviation from the non‐realistic GBLUP assumption of the infinitesimal model and improves the precision of estimates of SNP effects. In this procedure, the **H^−1^** matrix, calculated by combining all known pedigree and genotype information, is used in the ssGBLUP to estimate genomic estimated breeding values (GEBV) for all animals. Then, the GEBVs of the genotyped animals are used to estimate effects for the SNP. Finally, SNP effects are used to calculate the percentage of genetic variance explained by sets of consecutive SNP (SNP windows). In this study, the proportion of additive genetic variance explained by windows of 50‐adjacent SNP was calculated and the regions that accounted for more than 0.50% of the additive genetic variance were identified as potential QTL. The present study, identified three windows on BTA14 (in position 1.48–1.68, 1.85–2.11 and 2.67–2.94 Mb, respectively) in both primi‐ and multiparous cows associated with 305‐day milk yield or lactation curve parameters. The identified regions overlap with QTL regions for multiple traits including milk yield and milk composition, somatic cell count, calving ease and average daily gain in cattle (Bennewitz et al., 2003; Boichard et al., 2003; Buitenhuis et al., 2014; Iso‐Touru et al., 2016; Jiang et al., 2010; Lund et al., 2008; Nayeri et al., 2016; Rupp & Boichard, 2003). Iso‐Touru et al. (2016) identified 755 SNP in six different chromosomes (BTA5, BTA14, BTA16, BTA19, BTA20 and BTA25) associated with milk yield in Nordic Red cattle with the highest number of significant SNP on BTA14. Nayeri et al. (2016) using a single SNP regression mixed linear model, identified 292 SNP associated with milk yield in Canadian Holsteins, with the highest number of significant SNP on BTA14. Meredith et al. (2012), using a single SNP regression approach, identified 370 SNP associated with milk yield in Irish Holsteins with the highest number of significant SNP on BTA14.

A region on BTA14 in position 1.85–2.11 Mb was identified to be associated with 305‐day milk yield, peak yield and scale in both primi‐ and multiparous cows. This region contains several genes including *MIR2309, MIR1839, OPLAH, HGH1, GRINA, PARP10, MAF1, SHARPIN, CYC1, GPAA1, MROH1, EXOSC4* and *SPATC1*. The association of genes including *OPLAH, GRINA* and *MF1* with milk yield and lactation performance has been reported in previous studies (Kolbehdari et al., 2009; Nayeri & Stothard, 2016; Wang, Ning, Liu, Zhang, & Jiang, 2019); however, the association of the remaining identified genes inside this region with lactation performance in dairy cows has not been reported previously.

A region on BTA14 in position 1.48–1.68 Mb was identified to be associated with 305‐day milk yield, peak yield, scale and decay in primiparous cows. This region was also associated with 305‐day milk yield, peak yield and the scale in multiparous cows. Previous studies have reported this region to be associated with milk fat yield and milk protein percentage (Bagnato et al., 2008; Rodriguez‐Zas, Southey, Heyen, & Lewin, 2002). This region contains several genes including *MIR2308, CYHR1, FOXH1, COMMD5, TONSL, PPP1R16A, MFSD3, LRRC24, C14H8orf33, RECQL4, ARHGAP39, RPL8, GPT, LRRC14, ZNF34, C14H8orf82, ZNF7* and *KIFC2.* The association of genes including *TONSL, PPP1R16A, FOXH1, ARHGAP39, CYHR1* and *ARHGAP39* with milk yield and milk composition has been reported in previous studies (Buitenhuis et al., 2014; Nayeri & Stothard, 2016; Ning et al., 2017; Wang et al., 2019). Nayeri et al. (2016) reported that highly significant SNP for milk yield in Canadian Holsteins were mapped inside genes including *CPSF1*, *DGAT1*, *TONSL*, *CYHR1*, *FOXH1* and *PPP1R16A*.

It is documented that cows which reach peak production later, produce more 305‐day milk, more milk at peak, and show a higher milk yield persistency (Saghanezhad et al., 2017). The only windows associated with 305‐day milk yield, peak yield, peak time and decay was identified on BTA14 in position 2.67–2.94 Mb. This region which contains several genes, including *PSCA, LY6K, THEM6, LYNX1, JRK, ARC, SLURP1, LY6D, GML* and *LYPD2,* was also associated with 305‐day milk yield and peak yield in multiparous cows. Buitenhuis et al. (2014) reported that the *GML* is associated with milk fat and protein percentage; however, the association of the remaining identified genes inside this region with lactation performance in dairy cows has not been reported before.

The decay parameter represents the rate of decline in milk production after peak and can be considered as a measure for lactation persistency. The genomic regions explaining variation in lactation persistency have been investigated in several GWAS (Kolbehdari et al., 2008; Nayeri et al., 2017). Nayeri et al. (2017) identified 83 SNP in four different chromosomes (BTA6, BTA13, BTA20 and BTA27) to be associated with lactation persistency (defined as the average of expected milk yield at day 280 in lactation compared with that at day 60 in lactation) in Canadian Holsteins with the highest number of significant SNP on BTA20. Do et al. (2017) reported eight SNP on BTA2, 5, 9, 14, 19 and 20 to be associated with lactation persistency in Canadian Holsteins. However, the present study identified two regions (BTA14 position 1.48–1.68 Mb and 2.67–2.94 Mb) associated with decay parameter which has not been previously reported to be associated with lactation persistency in dairy cattle.

The square of the correlation coefficient between two loci (*r* ^2^) was used to map LD in the identified windows. In total, four LD blocks were found in the region identified on BTA14 in position 1.48–1.68 Mb. These strong LD blocks reflect the strong selection pressure towards allele fixation that has been carried out in this part of the bovine genome (Khatkar et al., 2008; McKay et al., 2007). The C‐terminal protein lipidation (GO: 0006501), C‐terminal protein amino acid modification (GO: 0018410) and protein lipidation (GO: 0006497) were identified as the enriched biological process terms. The protein lipidation, the covalent attachment of lipid groups to an amino acid in a protein, and C‐terminal protein amino acid modification, the alteration of the C‐terminal amino acid residue in a protein, are child terms of the C‐terminal protein lipidation, the covalent attachment of a lipid group to the carboxy‐terminus of a protein. Protein lipidation is an important co‐ or post‐translational modification which occurs in many proteins in eukaryotic cells and regulates numerous biological pathways such as membrane trafficking, protein secretion, signal transduction and apoptosis (Chen, Sun, Niu, Jarugumilli, & Wu, 2018; Jiang et al., 2018). Protein lipidation is essential for binding and partitioning in different membrane microdomains, and for the interaction with effectors and the regulation of signalling processes, thereby playing a key role in controlling protein localization and function (Triola, 2011).

## 5 CONCLUSION

The objective of this study was to identify genomic regions associated with milk yield and the shape of lactation curve in Holstein dairy cows. The lactation curve parameters, the slope of the initial rise of the curve, peak yield, time to peak and the slope of the curve after peak yield were used as new phenotypic variables in the GWAS. Among the parameters of lactation curve, scale and peak yield showed the highest heritability and the highest genetic correlation with 305‐day milk yield which can explain the overlapping regions among these traits. The genomic regions were found to be associated with 305‐day milk yield, scale and peak yield in both primi‐ and multiparous cows. Although during the last decade many animals have been genotyped using high‐density SNP chip panels, no significant impact has been observed on genetic improvement programme (Erbe et al., 2012; VanRaden et al., 2013). However, Abo‐Ismail et al. (2017) reported that combining the significant SNPs or SNPs within or nearby gene(s) from the HD panel with the BovineSNP50 panel yielded a marginal increase in the accuracy of prediction of genomic estimated breeding values compared to the use of the BovineSNP50 panel alone.

## CONFLICT OF INTEREST

The authors declare that they do not have any conflicts of interest.

## Data Availability

Relevant information supporting the results not presented in the manuscript is given in additional files (Data S1–S12).
